# Preparation, Characterization, and In Vivo Pharmacokinetic Evaluation of Polyvinyl Alcohol and Polyvinyl Pyrrolidone Blended Hydrogels for Transdermal Delivery of Donepezil HCl

**DOI:** 10.3390/pharmaceutics12030270

**Published:** 2020-03-16

**Authors:** Santosh Bashyal, Chang Yell Shin, Sang Min Hyun, Sun Woo Jang, Sangkil Lee

**Affiliations:** 1 College of Pharmacy, Keimyung University, 1095 Dalgubeol-daero, Dalseo-gu, Daegu 42601, Korea; bashyal.santosh18@gmail.com; 2 Research Institute of Dong-A ST Co. Ltd., Yongin 17073, Korea; pharm91@donga.co.kr (C.Y.S.); hsm19@donga.co.kr (S.M.H.)

**Keywords:** transdermal delivery, hydrogel, skin permeation, donepezil, propylene glycol, chemical enhancer

## Abstract

Transdermal delivery systems are emerging platforms for the delivery of donepezil hydrochloride (DH) for treating Alzheimer’s disease. The primary aim of this study was to develop polyvinyl alcohol and polyvinyl pyrrolidone blended hydrogels and to evaluate their feasibility for delivering DH via a transdermal route. Physicochemical properties, such as gel fraction (%), swelling ratio (%), weight loss (%), mechanical strength, elongation at break, and Young’s modulus of the prepared hydrogels were evaluated. Furthermore, in vitro skin permeation and in vivo pharmacokinetic studies were performed. With an increased concentration of propylene glycol (PG), the gel fraction (%), maximum strength, and elongation at break decreased. However, the swelling ratio (%) and weight loss (%) of hydrogels increased with increased PG content. The 26% PG-hydrogel was superior, with an enhancement ratio of 12.9 (*** *p* < 0.001). In addition, the 11% PG-hydrogel and 1% PG-hydrogel exhibited an enhancement ratio 6.30-fold (*** *p* < 0.001) and 2.85-fold (* *p* < 0.05) higher than that exhibited by control, respectively, indicating a promising effect of PG on skin permeation. In addition, in vivo pharmacokinetic studies on hairless rats assessed the expediency for transdermal delivery of DH. The transdermal delivery of optimized hydrogel-patches with two different doses of DH revealed that the maximum plasma concentration and area under the curve were dose dependent, and the time to reach the maximum concentration was 8 h. Thus, optimized hydrogels have the potential to enhance the transdermal delivery of DH and could be a novel clinical approach.

## 1. Introduction

Alzheimer’s disease (AD) is a chronic neurodegenerative disorder that causes dementia [1]. In 2015, 46.8 million people were affected by AD, and this prevalence is estimated to increase to 75 million and 131.5 million globally by 2030 and 2050, respectively [2]. There are approximately 5.8 million Americans with AD in 2019 [3]. Due to the impairment of cholinergic neurotransmission, there is a decreased production of the neurotransmitter acetylcholine, leading to AD [4]. Donepezil hydrochloride (DH) is the second well-tolerated FDA-approved medication for the treatment of AD [5]. DH is a centrally acting reversible acetylcholinesterase inhibitor that decreases the availability of beta amyloid and increases the concentration of acetylcholine [6,7]. DH is administered to patients orally; however, significant variations in DH plasma concentrations and gastrointestinal side-effects, including nausea, vomiting, anorexia, and abdominal pain, are associated with oral administration. In addition, owing to dementia and dysphagia, elderly patients have difficult in consuming medicines effectively [8,9]. Thus, transdermal drug delivery systems (TDDSs) can circumvent issues associated with oral administration and may be the best therapeutic platform for improving patient compliance.

TDDS offers a multitude of advantages over oral administration. Owing to the maintenance of constant plasma levels over a long period, the dosing frequency is attenuated and bioavailability of the drug across the skin is improved. Additionally, the first-pass effect and gastrointestinal discomfort can be avoided. Patients can easily terminate the treatment, which improves patient compliance [10,11,12]. The molecular structure and essential properties of DH are summarized in Table 1. All parameters required to increase lipophilicity and enhance drug permeability, such as molecular weight, H-bond donation, H-bond acceptance, and the log P value of DH, follow the Rule of Five. However, owing to the presence of a permeability barrier (the stratum corneum) in the skin, formulating a TDDS of DH is still a major concern for researchers [13,14]. Chemical permeability enhancers offer tremendous potential to overcome this limitation. In general, chemical enhancers facilitate the transfer of drug across skin layers mainly by two mechanisms: by extract of lipids directly from the skin (lipid extraction) and by partitioning into lipid bilayers (lipid fluidization) [15].

In this study, we used propylene glycol (PG) as the primary chemical permeation enhancer for facilitating DH permeability across the skin, both in vitro and in vivo. PG is reportedly a safe and effective chemical permeability enhancer across the skin [16]. PG is listed as generally regarded as safe by the United States Food and Drug Administration (FDA). In addition, the World Health Organization has reported that the acceptable daily intake of PG is up to 25 mg/kg body weight [17]. The mechanism of PG that facilitates drug permeability is not clearly understood. However, it has been proposed that PG acts by solvating the keratin present in the stratum corneum and intercalating the polar head groups of lipid bilayers [18]. 

Hydrogels are cross-linked three-dimensional hydrophilic polymeric networks and have the potential to retain substantial amounts of aqueous fluids or water [19,20]. Hydrogels have been widely explored for numerous biomedical applications, such as in drug delivery, soft contact lenses, biomaterials, artificial organs, superabsorbent implants, stimuli-responsive systems, wound dressings, or tissue engineering, owing to their excellent biocompatibility, biodegradability, non-toxicity, non-antigenicity, elasticity, high water content, and soft consistency [21,22]. These characteristics render hydrogels ideal for transdermal applications. The choice of polymers used to fabricate hydrogels has a significant impact on swelling, elasticity, and mechanical properties, as well as skin permeation of the drug. It was previously reported that poly(vinyl alcohol) (PVA) and poly(vinyl pyrrolidone) (PVP) are easily blended and cross-linked to form desirable hydrogels [23,24]. In addition, hydroxyl group of PVA will interact with carbonyl group of PVP leading to the formation of interchain of hydrogen bonding and enhances the network stability [25]. The drug will be located across this polymer network. However, the addition of cosolvent will reduces the cross-linking density and network formation resulting in the enhanced elasticity and flexibility of hydrogels. Moreover, cosolvent will engross the water molecules from the release medium, and increases the solubilization of the drug, leading to decreasing the viscosity of the hydrogels and increasing the release rate of the drug [26]. These PVA-PVP blended hydrogels have improved water uptake capacity, possess high elasticity and soft consistency, are pleasant to touch, terminate easily without pain, and have mechanical strength and transparency [27]. In this study, physical cross-linking techniques were adopted to cross-link PVA-PVP blended hydrogels owing to their superior qualities, including non-toxicity and higher mechanical strength, over chemical or irradiative methods [28].

The main objective of this study was to fabricate, optimize, and characterize PVA-PVP blended hydrogels for transdermal delivery of DH. In this perspective, the gel fraction (%), swelling ratio (%), weight loss (%), and mechanical properties of the developed hydrogels were evaluated, and the effect of PG on skin permeation of DH was assessed. Finally, in vivo pharmacokinetic studies were conducted using hairless rats to assess the expediency for transdermal delivery of DH.

## 2. Materials and Methods 

### 2.1. Materials

DH was provided by the Research Institute of Dong-A ST Co. Ltd. (Yongin, Korea). PVP K-90, PVP K-30, and PG were purchased from BASF (Ludwigshafen, Germany). PVA and glycerol were purchased from Sigma-Aldrich (St Louis, MO, USA). All other chemicals were of reagent-grade and used without further purification.

### 2.2. Preparation of Hydrogels

**Figure 1:**
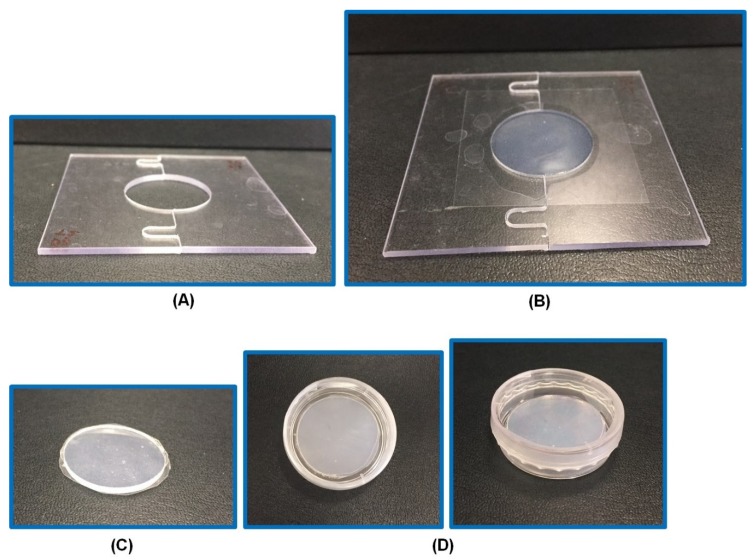
The specially designed mold for fabrication of circular hydrogels. A blank mold (**A**), a mold with an optimized hydrogel (26% PG [propylene glycol]-hydrogel) (**B**), 26% PG-hydrogel after its removal from the mold (**C**), 26% PG-hydrogel in a Petri dish sealed with parafilm (**D**).

PVA-PVP blended hydrogels were fabricated using a physical cross-linking method. First, each ingredient (PVA, PVP K-90, PVP K-30) was weighed, as indicated in Table 2. Then these ingredients were mixed in a beaker with the required amount of water. Subsequently, the beaker was sealed and heated for 25 min at 65–70 °C until a clear gel was obtained. To prevent the loss of water due to evaporation, the weight of the hydrogel was equalized with the initial water content and then the required amounts of PG and glycerol were added. Finally, DH was added and stirred for 5 min. The prepared hydrogel formulation was centrifuged at 2852× *g* for 5 min at 25 °C (Combi-514R; Hanil Science Industrial Co. Ltd., Gimpo, Korea) to remove air bubbles. After centrifugation, the prepared hydrogel was poured into a specially-designed mold, as shown in Figure 1, using a viscous liquid pouring pipette (Microman, Gilson^®^ pipette; Gilson Inc., Middleton, WI, USA) to minimize errors and to maintain constant weights of all the hydrogels. Afterward, the hydrogels in molds were chilled at 4 °C for 24 h and then thawed at room temperature for 2 h. Finally, the prepared hydrogels were stored in airtight Petri dishes until further use.

### 2.3. Determination of Gel Fraction (%)

To measure the gel fraction, circular hydrogel samples (2 cm diameter and 2 mm thickness) were prepared and then dried in an oven for 24 h at 50 °C (*W_o_*). After 24 h, dried hydrogel samples were immersed in 1000 mL distilled water for 24 h to reach equilibrium swelling and to remove the soluble parts. Subsequently, the samples were dried again in an oven at 50 °C (*W_e_*) for 24 h [26]. The gel fraction was calculated as follows:

(1) $$Gel\;fraction\;\left(\%\right)=\frac{W_{e}}{W_{o}}\times 100$$

### 2.4. Determination of Swelling Ratio (%)

To measure water uptake capacity, circular hydrogel samples (2 cm diameter and 2 mm thickness) were prepared and dried in an oven at 50 °C for 24 h. Then the weights of dried hydrogels (*W_e_*) were measured immediately. Subsequently, samples were immersed in distilled water at 37 °C for 24 h. Finally, the weight of the soaked hydrogel samples was measured (*W_s_*) [26]. The swelling ratio was calculated as follows:

(2) $$Swelling\;ratio\;\left(\%\right)=\frac{W_{s}-W_{e}}{W_{e}}\times 100$$

### 2.5. Weight Loss (%) 

To measure the rate of water evaporation, circular hydrogel samples (2 cm diameter and 2 mm thickness) were prepared, weighed (*W_o_*), and then stored in an oven at 37 °C. Subsequently, the weights of the samples were measured at different time points (*W_t_*) [29]. The percentage of weight loss was calculated as follows:

(3) $$Weight\;loss\;\left(\%\right)=\frac{W_{t}}{W_{o}}\times 100$$

### 2.6. Determination of the Mechanical Properties

To measure the mechanical tensile strength and elongation at break, prepared hydrogel samples were cut into a specific “dog bone” shape, i.e., 6 cm long, 1.6 cm wide at the ends, and 0.8 cm wide in the middle, and then evaluated using a ball screw-driven motorized test frame (Mecmesin Multitest 2.5i, Mecmesin, Sterling, VA, USA) equipped with a 50 N load cell. Subsequently, the samples were clamped between tensile grips. The initial distance between the grips was 20 mm, and the crosshead speed was 20 mm/min. The thickness of each hydrogel was also measured with a Vernier caliper. Tensile strength and breaking elongation at break were evaluated from stress–strain curves. Young’s modulus was also calculated as the slope of the linear line of the stress–strain curve [30,31].

### 2.7. In Vitro Skin Permeation Study

The skin preparation and in vitro skin permeation study were performed using our previous well-established method [32,33]. Briefly, the skin was obtained from male Sprague–Dawley rats (7–8 weeks old, 220–240 g). Rats were anesthetized using an intraperitoneal injection of urethane and the hair from the dorsum was removed with electric clippers. Subsequently, a depilatory was applied to detach remaining hair and then the skin was excised using surgical scissors. The underlying adipose tissue from the skin was carefully eliminated with scalpel and forceps and stored at −20 °C until further use.

The in vitro permeation study was performed using rat skin in a vertical static Franz diffusion cell with an effective diffusion area of 1.77 cm^2^. The skin tissue was fixed horizontally between the donor and receptor compartments. The hydrogel sample (equivalent to 37 mg DH) was then placed above the skin, and the study was performed from the donor to the receptor compartment of the Franz diffusion cell filled with phosphate-buffered saline (pH 7.4) with constant stirring using a magnetic stirrer at 600 rpm, maintained at 37 °C. A total of 0.5 mL of sample was withdrawn from the receptor compartment at pre-determined time points over 72 h and replaced immediately with an equal volume of phosphate-buffered saline (pH 7.4) to maintain a constant volume. Each experiment was performed in triplicate. 

The amount of permeated DH was determined by reverse-phase high-performance liquid chromatography (RP-HPLC) using a previously reported method [34]. Briefly, an Agilent 1200 infinity series LC system (Agilent Technologies Inc., Santa Clara, CA, USA) was used with an Agilent Eclipse XDB-C18 column (5 µm, 4.6 × 250 mm). The mobile phase was a mixture of methanol and 0.02 M monobasic sodium phosphate (60:40) with a flow rate of 1 mL/min and a UV detection wavelength of 268 nm. Phosphate buffer was prepared by dissolving monobasic sodium phosphate in distilled water, followed by adding 10 mL of triethylamine and adjusting the pH to 2.7 ± 0.5 with phosphoric acid. 

The steady-state flux (*J_s_*), permeability coefficient (*K_p_*), lag time, and enhancement ratio (ER) were calculated as described previously [32,35]. Briefly, *J_s_* was calculated as follows:

(4) $$J_{s}=\frac{Q_{r}}{A\cdot t}\left(\mathrm{mg}\cdot {\mathrm{cm}}^{-2}\cdot h^{-1}\right)$$

where *Q_r_*, *A*, and *t* are the total permeated DH (mg), diffusion area (cm^2^), and the time of exposure (h), respectively.

Similarly, *K_p_* was calculated as follows:

(5) $$K_{p}=\frac{J_{s}}{C_{d}}\left(\mathrm{cm}\cdot h^{-1}\right)$$

where *C_d_* is the initial concentration of DH (mg·cm^−3^). In addition, the lag time was calculated from the x-intercept of the linear regression line. Finally, ER was calculated by the ratio of the *K_p_* value of each hydrogel formulation to that of the control (0% PG-hydrogel).

### 2.8. Animals

Male, hairless, 6-week-old rats were obtained from Central Lab Animal Inc. (Seoul, Korea). Animals were maintained at (23 ± 2) °C under a 12 h light/dark cycle (lights on 07:00–19:00) and were provided with access to food and water ad libitum. All animal studies were approved by the Institutional Animal Care and Use Committee (IACUC: I-1903046, 14 March 2019) of the Research Institute of Dong-A ST.

### 2.9. Pharmacokinetic Study in Hairless Rats

To assess the pharmacokinetic profiles, hairless rats with an average weight of 450–500 g were randomly assigned to corresponding groups of five animals per group. The hairless rats were anesthetized with diethyl ester, patched, and dressed in adhesive stretch bandages for patch fixation. To evaluate dose effects, the optimized hydrogel (26% PG-hydrogel) was prepared with two different proportions of DH (5.85% and 11.7%). DH hydrogel-patches were applied transdermally to the back skin of hairless rats. Blood (250 µL) was collected from the tail vein at each indicated time point: 0, 8, 24, 30, 48, and 72 h. Similarly, DH (1 mg/kg) was administered intravenously, and the blood (250 µL) was collected from the tail vein at each indicated time point: 0, 0.25, 0.5, 1, 2, 4, 6, 8, and 24 h. These samples were kept at –80°C until analysis. 

### 2.10. Analysis of Plasma Donepezil Hydrochloride (DH) Levels

Plasma DH concentrations were analyzed using LC-MS/MS with a slight modification of a previously reported method [36,37]. Briefly, DH (10 mg) was accurately weighed into a 20 mL volumetric vial and dissolved in methanol to prepare a working stock solution of 1000 µg/mL. An aliquot (100 µL) of working stock solution (1000 µg/mL) was transferred to a 1 mL E-tube and serially diluted with methanol to obtain working solutions ranging from 78 to 1000 ng/mL. Depending on the calibration range and matrix (plasma) used, samples were prepared in the following manner. A suitable aliquot of samples or control matrix was accurately pipetted into a 2 mL tube and spiked with the internal standard solution (amantadine 250 ng/mL; 300 µL). For calibration and quality control (QC) samples, a suitable volume of calibration and QC spiking solution was added. Standards were diluted to a final concentration ranging from 3.9 to 500 ng/mL. All samples were analyzed using LC-MS/MS with an Agilent Technologies 1200 series HPLC system coupled to a 6430 Triple Quad LC/MS (Santa Clara, CA, USA). Chromatography was performed on a Union UK-C18 3µ column (50 × 2.0 mm; Portland, OR, USA). A sample aliquot of 5 µL was injected onto the LC-MS/MS system with an auto-sampler followed by a needle wash using methanol. A gradient HPLC system (mobile ratio [A:B] = 25:75) was used with mobile phases (A) formic acid (0.05%), and (B) methanol (100%) at a total flow rate of 0.2 mL/min. Data were obtained using proprietary software from the instrument manufacturer. Peak areas and quantitative data were generated using MassHunter Workstation Software Quantitative Analysis Version B 04.00 (Agilent Technologies; Santa Clara, CA, USA). To compare the bioavailability of transdermal hydrogel-patches of DH with the bioavailability of DH following intravenous administration, absolute bioavailability (F) was determined. F was calculated as follows:

(6) $$F=\frac{AUC_{transdermal}}{AUC_{IV}}\times \frac{Dose_{IV}}{Dose_{transdermal}}$$

### 2.11. Statistical Analysis

The results are expressed as the mean ± standard deviation. One-way analysis of variance followed by Tukey’s multiple comparison test was used to determine statistically significant differences between the groups. Results of *p* < 0.05 were considered statistically significant.

## 3. Results and Discussion

### 3.1. Preparation and Characterization of Hydrogels

Hydrogels are a cross-linked network of hydrophilic polymers. Hydrogels have received considerable attention among drug delivery scientists, medical doctors, biomaterials scientists, and tissue engineers for various biomedical applications [38]. The selection of polymer is crucial for the fabrication of hydrogels. We prepared various hydrogels with PVA and two different grades of PVP (K-90, K-30) using a physical cross-linking method. This method involves simple freeze-thawing steps. In this study, we incubated a PVA-PVP blended hydrogel for 24 h at 4 °C as a freezing step, followed by 2 h thawing at room temperature. This method overcomes toxicity issues and poor mechanical strength attributed to chemical and irradiation methods [28]. We used PG as a solubilizer and a chemical enhancer. We screened and prepared various hydrogels with PVA, PVP, and PG with DH at different percentages. We observed that 7.5% PVA, 10% PVP, and 26% PG showed optimal morphological characteristics, i.e., transparency, elasticity, gel formation, adhesiveness, and mechanical properties. Further, we optimized the percentage of PVP K-90 and PVP K-30 to be 8% and 2%, respectively, based on the adhesive properties, as we observed that PVP K-90 imparts higher adhesiveness to the hydrogel than PVP K-30. After 2-3 days, there was leakage of liquid from the hydrogel. Thus, to surmount this problem, 1% glycerol (a humectant) was added. Finally, the optimized hydrogel was 26% PG-hydrogel (Figure 1). The organoleptic property of optimized hydrogel is presented in Table 3.

#### 3.1.1. Determination of the Gel Fraction (%)

**Figure 2:**
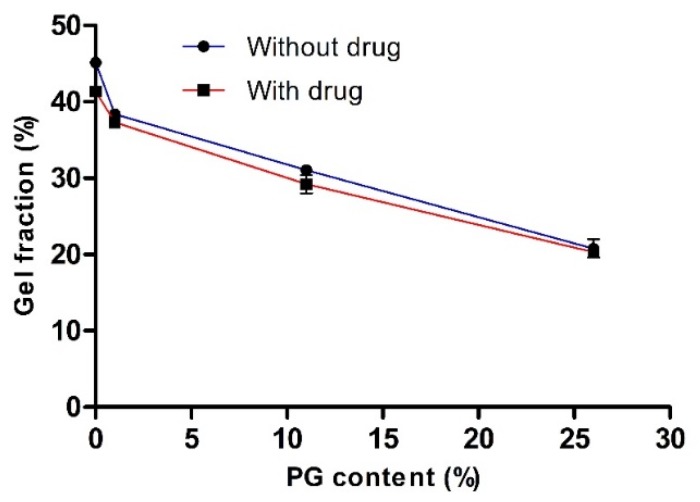
The effect of propylene glycol (PG) on the gel fraction (%). Data are expressed as the mean ± standard deviation (*n* = 4).

To evaluate the effect of PG on cross-linking density and network formation, we measured gel fractions of various hydrogels with or without the drug. The influence of PG on the gel fraction (%) is illustrated in Figure 2. This study revealed that the gel fraction (%) increased as the concentration of PG decreased. In the absence of both the drug and PG and the presence of the drug (with drug and 0% PG), the gel fraction (%) was increased to the maximum, and the value was 45.13 ± 0.46 and 41.33 ± 0.14, respectively. There was no significant difference in gel fraction (%) with or without the drug. This finding confirmed that PVA-PVP was completely cross-linked and entangled. It has been reported that PVA and PVP are readily blended and cross-linked in their homogeneous mixture with water [24].

Nevertheless, the gel fraction (%) was reduced with increased PG content and the value reached to 20.77 ± 1.19 and 20.29 ± 0.12 at 26% PG content, in the absence and presence of the drug, respectively. This finding may be because, in the presence of drug and PG, the cross-linking density may decrease, resulting in fewer entanglement reactions in the PVA-PVP hydrogel. Consequently, the cross-linking network chains are reduced by reducing physical interactions between the PVA and PVP network and, thus decreased the gelation process. These results were in accordance with the observation of previously obtained results [26,39,40]. Harthi et al. recently studied the gelation behavior of PVP hydrogels with different contents of polyethylene glycol (PEG) and found that the gel fraction (%) was reduced as the concentration of PEG increased, while the gel fraction (%) was maximum at 0% PEG content (PVP hydrogel without PEG content) [26]. In addition, the gelation behavior of PVA hydrogel was maximal, almost 90%, in the absence of sodium alginate (SA) and decreased with the increasing content of SA, i.e., a 27% gel fraction at 30% SA [39].

#### 3.1.2. Determination of the Swelling Ratio (%)

**Figure 3:**
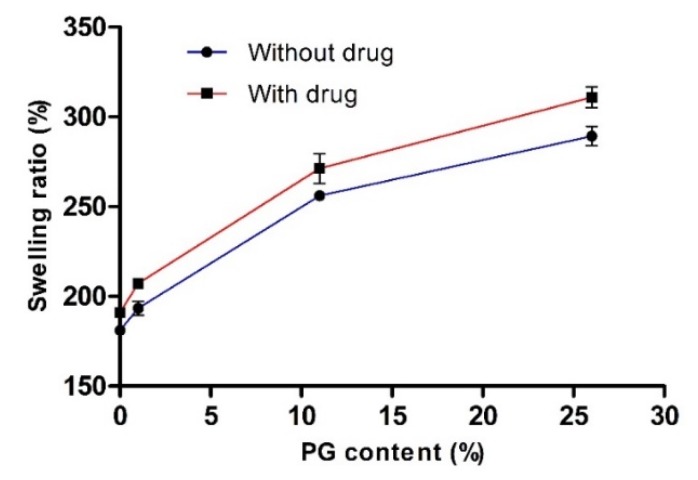
Effect of propylene glycol (PG) on the swelling ratio (%). Data are expressed as the mean ± standard deviation (*n* = 4).

To investigate the influence of PG on water engrossment capacity, we measured the swelling ratio (%) of various hydrogels with or without the drug. The effect of PG on the swelling ratio (%) is presented in Figure 3. This data clearly showed that the swelling ratio (%) was increased with increasing concentration of PG. In the absence and presence of the drug (without drug and 26% PG; with drug and 26% PG), the swelling ratio (%) was increased to the maximum and the value reached 289.32 ± 5.36 and 310.89 ± 5.82, respectively. There were no significant differences in the swelling ratio (%) with or without the drug. This finding may be attributed to the PG content, as PG is a hydrophilic and hygroscopic molecule, which results in uptake of a large amount of water and does not take part in the cross-linking network. In general, cross-linking density has an inverse relationship with the swelling ratio [41]. However, in the absence and presence of drug (without drug and 0% PG; with drug and 0% PG), the swelling ratio (%) was reduced with the decreased PG content, with values reaching 181.11 ± 2.73 and 191.11 ± 2.36 at 0% PG content, respectively, resulting in high cross-linking density of PVA-PVP blended hydrogels. Consequently, this network formation takes up small amounts of water.

These results were consistent with previously reported studies [26,39,40]. Kamoun et al. studied water uptake (%) behavior of PVA hydrogels with and without drug and found that the uptake ratio (%) with drug was maximized with a hydrogel containing 75% SA (4200%), while at 0% SA content, the uptake ratio (%) decreased to 1500%. It has been reported that higher SA concentrations enhanced the wettability and hydrophilic nature of hydrogels, resulting in a higher swelling ratio [40]. In another study, the swelling ratio of a PVP hydrogel with 3% PEG revealed higher swelling capacity than pure PVP hydrogel (0% PEG). The authors attributed this finding to the PEG content, as PEG is hydrophilic and drives swelling. This study concluded that with increased PEG content, the swelling ratio of PVP hydrogels increased gradually [26].

#### 3.1.3. Determination of Weight Loss (%)

**Figure 4:**
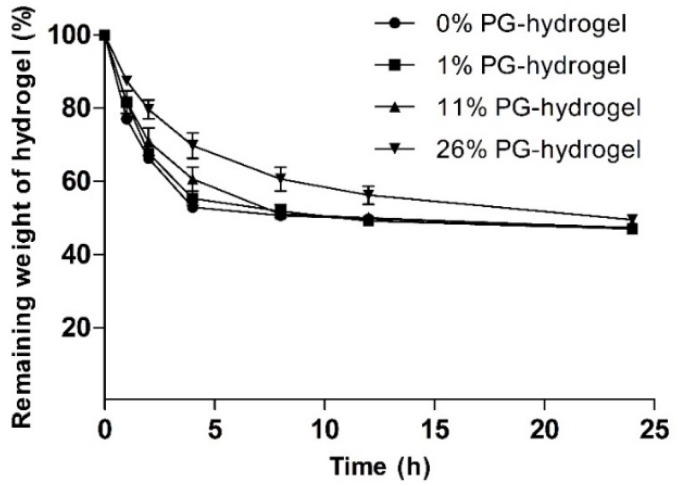
Effect of propylene glycol (PG) on weight loss (%). Data are expressed as the mean ± standard deviation (*n* = 3).

To evaluate the effect of PG on the water evaporation rate, we measured the weight loss (%) of various hydrogels. The influence of PG on weight loss (%) is illustrated at different time intervals in Figure 4. This figure shows that the water loss from hydrogels increased gradually with time. It was found that the remaining weight (%) of the control hydrogel, 1% PG-hydrogel, 11% PG-hydrogel, and 26% PG-hydrogel for the first 2 h were 66.23 ± 0.67, 67.64 ± 1.12, 70.84 ± 3.80, and 79.62 ± 2.57, respectively. As time passed, water loss gradually increased, and at certain times, it remained constant. After 8 h, the remaining weight of the control hydrogel, the 1% PG-hydrogel, and the 11% PG-hydrogel were nearly identical, with an approximate water loss of 50%. However, the remaining weight (%) of the 26% PG-hydrogel was 60.60 ± 3.23, i.e., the water loss was 40%. It was observed that there was a slower loss of water from the hydrogel containing PG, and the loss of water was dependent upon PG content. This decreased loss is because PG is hydrophilic and hygroscopic. Finally, after 12 h and 24 h, the remaining weight (%) of the optimized hydrogel (26% PG-hydrogel) was 56.23 ± 2.47 and 49.47 ± 1.18, respectively. The loss of water from marketed dressings such as Geliperm^®^ (Geistlich Ltd., Wolhusen, Switzerland) is approximately 50% after 12 h [42]. Thus, our optimized hydrogel (26% PG-hydrogel) showed similar behavior, i.e., after 12 h and 24 h, the loss of water from the optimized hydrogel was approximately 44% and 51%, respectively. These results were also compatible with previously reported results by Balakrishnan et al. [29].

#### 3.1.4. Mechanical Properties Determinations

**Figure 5:**
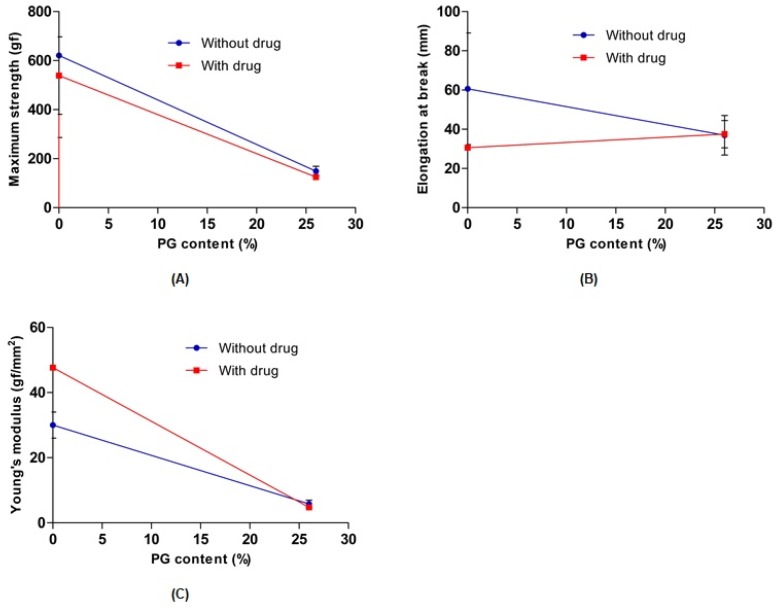
Effect of propylene glycol (PG) on the maximum strength (**A**), elongation at break (**B**), and Young’s modulus (**C**). Data are expressed as the mean ± standard deviation (*n* = 3).

To evaluate the effect of PG on the mechanical properties of 0% PG-hydrogel and 26% PG-hydrogel samples, their tensile strength, elongation at break, and Young’s modulus were measured. In the absence and presence of the drug, the maximum strength, elongation at break, and Young’s modulus of the hydrogel decreased with increasing PG contents. However, the elongation at break of both hydrogels containing the drug remained similar to increasing content of PG. Overall, the results revealed that PG affected the cross-linking density of the hydrogels and weakened the breaking elongation, resulting in decreased mechanical strength as well as Young’s modulus (Figure 5). These results are consistent with previously published reports [40,43]. The maximum tensile strength and elongation at break were sharply increased with the PVA hydrogel (0% SA) and decreased with increasing concentration of SA [39].

### 3.2. In Vitro Skin Permeation Study

**Figure 6:**
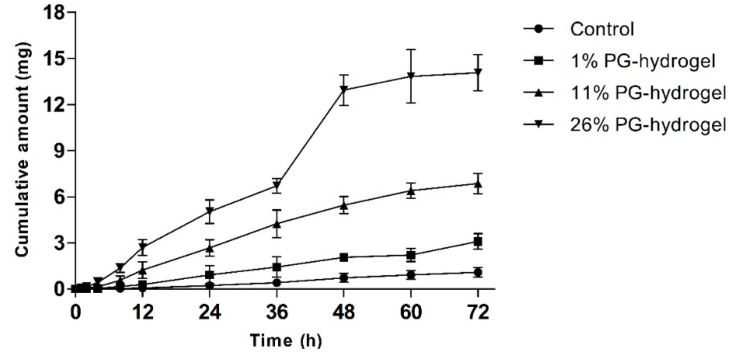
In vitro rat skin permeation by donepezil hydrochloride (DH). The effect of propylene glycol (PG) on transdermal delivery of DH. Error bars represent standard deviation (*n* = 3).

To evaluate whether PG affected the permeation profiles of DH, we performed an in vitro skin permeation study with various hydrogels for 72 h. The cumulative permeation profiles of DH-loaded hydrogels through rat skin are illustrated as a function of time (Figure 6). The cumulative amount permeated by the 26% PG-hydrogel was approximately 13 times greater than that of the control hydrogel, 4.5 times greater than that of the 1% PG-hydrogel, and twice greater than that permeated by the 11% PG-hydrogel. Alike to Corplex^TM^ Donepezil (investigational transdermal formulation), the total amount permeated by the 26% PG-hydrogel at 24 h was 4.69 mg, approximately similar, aiming to deliver 5 mg per day over the period of 72 h [44]. The skin permeation parameters *J_s_*, *K_p_*, and ER were calculated and are summarized in Table 4. The rise of these parameters was observed in the order of 26% PG-hydrogel > 11% PG-hydrogel > 1% PG-hydrogel > control (0% PG-hydrogel). The 26% PG-hydrogel revealed the highest *J_s_* and *K_p_*, which were significantly greater than those of other hydrogel formulations (*** *p* < 0.001 vs. control; ### *p* < 0.001 vs. 1% PG-hydrogel and $$$ *p* < 0.001 vs. 11% PG-hydrogel). Similarly, the *J_s_* and *K_p_*, for 11% PG-hydrogel were also significantly higher than the control hydrogel (*** *p* < 0.001) and the 1% PG-hydrogel (### *p* < 0.001). In addition, the *J_s_* and *K_p_* for the 1% PG-hydrogel were also significantly greater than that of the control hydrogel (* *p* < 0.05). The lag time of DH permeation was reduced 5.97 times by 26% PG-hydrogel compared to the control hydrogel. The 26% PG-hydrogel had a higher ER (12.9-fold) than the control hydrogel. In addition, the 11% PG-hydrogel and 1% PG-hydrogel also exhibited ER values 6.30-fold and 2.85-fold higher than that of the control, respectively. 

The permeation of the drug across the skin is greatly influenced by the polymer and the solvent used in the hydrogel formulation. The cross-linked network of a hydrogel is enhanced with the addition of PVA and PVP (K-90, K-30), and consequently decreases the elasticity and flexibility of hydrogels. However, the cross-linking density and network formation diminished with the incorporation of PG into the hydrogel, and thus, the elasticity and flexibility will be enhanced [26]. This behavior might be one crucial factor in increasing the skin permeation of DH. PG is a hygroscopic as well as a hydrophilic molecule [17]. It engrosses the water molecules from the receiver medium. This study revealed that 26% PG-containing hydrogel had the highest skin permeation of DH compared to the other hydrogels, and the hydrogels followed the concentration-dependent trend of PG, i.e., permeated amounts increased with increasing concentrations of PG. This finding is because PG acts as a solubilizing cosolvent, a permeation enhancer and has the potential to solvate the keratin of the stratum corneum [15]. Consequently, PG penetrates the skin tissues and occupies the hydrogen bonding sites leading to intracellular transport of drug and enhanced skin permeation [45]. These results were in agreement with previous reports [46,47]. PG has the potential to intercalate into the polar head groups of the lipid bilayer due to its hydrophilic nature and significantly enhances the mean interfacial area per lipid [48]. Moreover, skin partitioning and the barrier function of the skin are decreased by enhanced trans-epidermal water loss and kallikrein 7 (KLK 7) protease activity, which might account for the enhancement of skin permeation [49,50]. Yamane et al. reported that the flux and permeability coefficient of 5-fluorouracil was increased with the addition of terpenes to the PG system. The activity of terpenes was dependent upon the PG concentration, and the flux was maximized with a formulation containing 80% PG, which demonstrates the concentration-dependent relationship between flux and PG content. It was concluded that the drug partitioning and lipid disruption are the main mechanisms, resulting in enhanced skin permeation across human epidermis [46]. 

In another study performed by Lee et al., PG acted as an enhancer and increased the partitioning of PG into the stratum corneum, leading to the maximum flux of acetaminophen with 30% PG content, compared with a 10% PG formulation, which in turn was higher than a 0% PG-containing formulation [47]. Furthermore, the in vitro percutaneous permeation of loperamide hydrochloride and PG in a formulation containing 15% and 40% PG was tested, and low amounts of drug and substantial amounts of PG were permeated; this confirmed time-dependent penetration of the drug and suggested that the mechanism of permeability involved depletion of PG on the skin surface. In addition, there was a dose-dependent correlation between PG content and permeated drug [51]. Recently, a monolithic transdermal patch system incorporated with nanostructured lipid carriers (NLCs) was developed to co-deliver olanzapine and simvastatin, and screened with various chemical enhancers such as PG, Transcutol^®^, menthol, or limonene to determine their skin permeation profiles. Among these, PG was the best enhancer for olanzapine and simvastatin with an ER of 8.05 and 12.89, respectively, when compared with the control (Combo-NLC patch without enhancer) [52]. Moreover, a molecular dynamics simulation study was performed to elucidate the mechanism and found that PG was intercalated on the hydrophilic region of the phospholipid bilayers and increased the lateral diffusion of phospholipid molecules, suggesting pronounced enhancement profiles due to increased fluidity by disruption of highly ordered lipid lamellae [52]. In addition, PG promoted the flux of metronidazole, triprolidine base, cinnamaldehyde, and ibuprofen [16].

### 3.3. Pharmacokinetic Profiles of the DH Hydrogel-Patch in Hairless Rats

**Figure 7:**
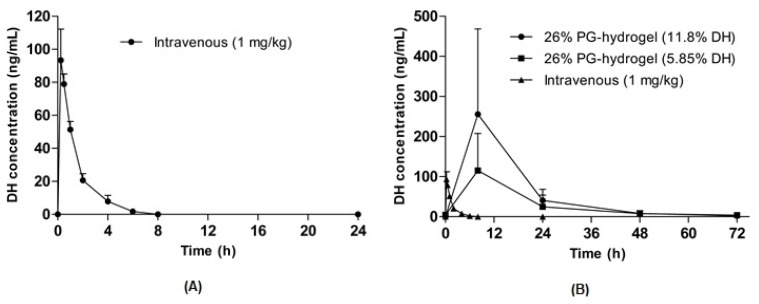
Pharmacokinetic profiles of donepezil hydrochloride (DH) in hairless rats. Intravenous injection of DH (1 mg/kg) (**A**) and the plasma concentration-time curve for 72 h after the application of DH hydrogel-patches (**B**). Data are expressed as the mean ± standard deviation (*n* = 5).

To evaluate the feasibility of optimized hydrogel formulations, in vivo pharmacokinetic studies were performed in hairless rats after the transdermal application of DH hydrogel-patches. The plasma concentration-time profiles of DH following intravenous and transdermal administration are illustrated in Figure 7A,B. The pharmacokinetic parameters such as the time to reach the maximum concentration (T_max_), the maximum (or peak) plasma concentration (C_max_) and the area under the curve (AUC) were calculated and are summarized in Table 5. An intravenous bolus of 1 mg/kg was administered to hairless rats (Figure 7A), and the C_max_ and AUC were 93.4 ± 18.9 ng/mL and 133.4 ± 23.5 ng·h/mL, respectively. In addition, the C_max_ for DH was achieved at 8 h and other parameters were dose-dependent after the application of transdermal DH hydrogel-patches. The C_max_ of the 11.7% DH hydrogel-patch and the 5.85% DH hydrogel-patch were 255.4 ± 95.3 ng/mL and 115.2 ± 41.3 ng/mL, respectively. Similarly, the AUC of the 11.7% DH hydrogel-patch was nearly 2 times higher than that of the 5.85% DH hydrogel-patch, suggesting dose-dependent characteristics of hydrogel-mediated permeation. The comparison of transdermal administration with that of intravenous administration of the same drug is indicated by F. The F of the 11.7% DH hydrogel-patch and the 5.85% DH hydrogel-patch was determined using dose-normalized AUC values following oral and intravenous administration, indicating F itself depends on the dose and the rate of absorption. Thus, the 11.7% DH hydrogel-patch and the 5.85% DH hydrogel-patch had similar normalized value of F.

## 4. Conclusions

PVA-PVP blended hydrogels were developed using a physical cross-linking method. The entanglement of PG within the cross-linked network of PVA-PVP blended hydrogels notably influence its physicochemical properties such as gel fraction (%), swelling ratio (%), weight loss (%), mechanical strength, breaking elongation, Young’s modulus, and skin permeation, as well as in vivo pharmacokinetics profiles of DH. In summary, the application of this optimized hydrogel demonstrated a feasible alternative for transdermal delivery of DH, and thus, hydrogel-based TDDS could be a promising platform for delivery of DH and for the effective treatment of AD.

## Figures and Tables

**Table 1:** Molecular structure and essential properties of donepezil hydrochloride (DH).

Molecular Structure	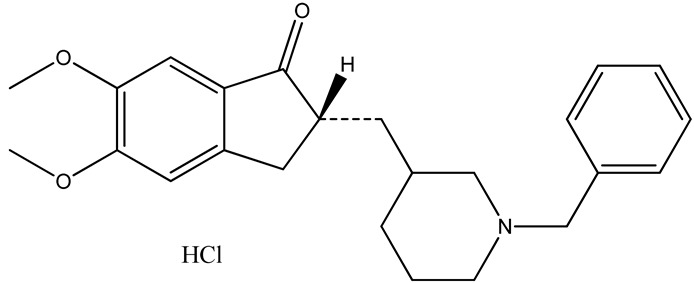
**Molecular weight**	415.96 g/mol
**H-bond donor**	1
**H-bond acceptor**	4
**Log P**	4.27
**pK**_a_	8.9

**Table 2:** Composition of prepared hydrogels (%, w/w).

Ingredients	0% PG-Hydrogel	1% PG-Hydrogel	11% PG-Hydrogel	26% PG-Hydrogel
PVA	7.5	7.5	7.5	7.5
PVP K-90	8	8	8	8
PVP K-30	2	2	2	2
PG	0	1	11	26
Glycerol	1	1	1	1
DH	11.7	11.7	11.7	11.7
Water	69.8	68.8	58.8	43.8
Total	100	100	100	100

**Table 3:** Characteristics of optimized hydrogels according to visual observation.

Observation (Organoleptic Property)
Gels form well
Homogeneous
Smooth texture
Transparency
Good flexibility
Medium adhesiveness
Medium mechanical strength
Medium elasticity
No drug crystallization
No leakage of liquid

**Table 4:** Skin permeation parameters for different hydrogel formulations. Data are expressed as the mean ± standard deviation (*n* = 3). * *p* < 0.05 vs. control, *** *p* < 0.001 vs. control; ## *p* < 0.01 vs. 1% PG [propylene glycol]-hydrogel, ### *p* < 0.001 vs. 1% PG-hydrogel; $$$ *p* < 0.001 vs. 11% PG-hydrogel.

Hydrogel	*J_s_* (mg·cm^−2·^h^−1^)	*K_p_* (cm/h) × 10^−4^	Lag Time (h)	Enhancement Ratio (ER)
Control	0.009 ± 0.002	2.32 ± 0.65	3.76 ± 0.66	1.00
1% PG-hydrogel	0.024 ± 0.004 ^*^	6.60 ± 1.08 ^*^	2.10 ± 3.05	2.85
11% PG-hydrogel	0.054 ± 0.005 ^***,^^##^	14.62 ± 1.40 ^***,^^##^	0.23 ± 2.67	6.30
26% PG-hydrogel	0.110 ± 0.009 ^***,###,$$$^	29.95 ± 2.49 ^***,###,$$$^	0.63 ± 1.04	12.90

**Table 5:** Donepezil hydrochloride (DH) pharmacokinetic parameters after intravenous (IV) injection or application of DH hydrogel-patches to hairless rats. Data are expressed as the mean ± standard deviation (*n* = 5).

Parameter	IV	DH Hydrogel-Patch	
Dose	1 mg/kg	5.85%	11.7%
T_max_ (h)	-	8.0 ± 0.0	8.0 ± 0.0
C_max_ (ng/mL)	93.4 ± 18.9	115.2 ± 41.3	255.4 ± 95.3
AUC_0__→last_ (ng·h/mL)	133.4 ± 23.5	1815.1 ± 631.9	3420.3 ± 1087.6
F	-	0.35	0.33
